# Neoadjuvant Chemotherapy Improves the Immunosuppressive Microenvironment of Bladder Cancer and Increases the Sensitivity to Immune Checkpoint Blockade

**DOI:** 10.1155/2022/9962397

**Published:** 2022-07-21

**Authors:** Hao Luo, Gao-Lei Liu, Dan Jian, Dan-Dan Liang, Xue-Mei Li, Li Zhong, Bo Yang, Jun Jiang, Dong Wang, Meng-Xia Li, Wei-Hua Lan, Nan Dai

**Affiliations:** ^1^Department of Oncology, Army Medical Center, Chongqing 400042, China; ^2^Department of Urology, Army Medical Center, Chongqing 400042, China

## Abstract

Although tumor immune microenvironment plays an important role in antitumor therapy, few studies explored the gene signatures associated with the tumor immune microenvironment of bladder cancer after neoadjuvant chemotherapy. We examined and analyzed differentially expressed genes from 9 patients with stage I-III bladder cancer by RNA immune-oncology profiling platform. After neoadjuvant chemotherapy, the expressions of 43 genes in 19 pathways and 10 genes in 5 pathways were upregulated and downregulated, respectively. Neoadjuvant chemotherapy also promoted the expression of genes related to the activation of antitumor immune responses and decreased the expression of genes related to tumor proliferation pathways. In addition, neoadjuvant chemotherapy improved tumor response to immune checkpoint blockade. Furthermore, this study also identified several genes that can be used to predict the efficacy of neoadjuvant chemotherapy and their possible molecular mechanisms. In conclusion, neoadjuvant chemotherapy may promote the activation of antitumor effects, improve the suppressive tumor immune microenvironment, and increase the sensitivity of bladder cancer to immune checkpoint blockade.

## 1. Introduction

Bladder cancer is one of the top ten most common cancer types in the world. In 2018, there were approximately 82,270 newly diagnosed bladder cancer cases in China, with an incidence rate of 5.8 cases per 100,000 people [1]. According to whether the cancer cells invade muscularis propria, bladder cancer can be classified into nonmuscle invasive bladder cancer (NMIBC) and muscle invasive bladder cancer (MIBC). Among them, NMIBC accounts for the majority of newly diagnosed bladder cancer cases. Despite the low incidence of MIBC, nearly 50% of MIBC patients will relapse after radical cystectomy [2]. The median age of patients diagnosed with bladder cancer is 73 years, and radical cystectomy can affect the patient's tolerance to chemotherapy. A retrospective analysis of 1,143 patients who underwent radical cystectomy found that more than 30% of patients experienced grade 2-5 complications within 90 days after surgery, leading to delays or intolerance to receive adjuvant chemotherapy [3]. Although the major guidelines in clinical practice recommend cisplatin-based chemotherapy for pT3/4 or lymph node-positive bladder cancer and adjuvant chemotherapy for MIBC, they are currently not supported by level 2A evidence-based medicine. The results of randomized controlled studies support that cisplatin-based neoadjuvant chemotherapy can improve the long-term survival rate of bladder cancer [4–6]. In the SWOG-8710 trial, the 5-year survival rate of patients who achieved pathological complete response after neoadjuvant chemotherapy reached 85% [6]. In addition, a meta-analysis showed that in the neoadjuvant chemotherapy regimen containing cisplatin, the 5-year overall survival rate increased by 5% and the risk of death reduced by 14% [7]. Therefore, based on these high-level evidences, the main guidelines recommend a cisplatin-based neoadjuvant chemotherapy regimen as the standard perioperative treatment of MIBC [8].

Tumor growth in the microenvironment is a complex process that includes the interplay between epithelial and stromal cell activation, vascular proliferation, inflammatory, and immune cell activation [9]. Under normal circumstances, T lymphocytes can recognize abnormal malignant cells, activate cytotoxic T lymphocytes through helper T cells, and infiltrate and kill these malignant cells. However, malignant cells have also developed complex mechanisms and pathways to block the activation of cytotoxic T cells and negatively regulate T cell activity, thereby conferring tumor immune escape [10]. Many studies have found that cell subsets in the immune microenvironment can be used as biomarkers for cancer prognosis and therapeutic efficacy [11], and chemotherapy can induce immunogenic cell death, release tumor-associated neoantigens, and trigger immune activation [12–14]. Recent studies have also found that neoadjuvant chemotherapy can even change the immune microenvironment of non-small-cell lung carcinomas [12].

However, there are few studies on the effect of neoadjuvant therapy on the immune microenvironment of bladder cancer. It is not yet clear which immune indicators can be used to predict the prognosis of bladder cancer after treatment. The aims of this retrospective study were to investigate the impact of neoadjuvant chemotherapy on the immune microenvironment of patients with bladder cancer and to explore the predictive value of the immune-related gene expression after neoadjuvant chemotherapy on the prognosis of bladder cancer.

## 2. Materials and Methods

### 2.1. Patients

From April 2018 to April 2019, a total of 24 patients were collected and 9 patients who underwent neoadjuvant therapy with stage II-III urothelial bladder carcinoma from Daping Hospital (i.e., Army Medical Center) were included in this retrospective cohort. The clinical data including patients' clinical and pathological characteristics, treatment, and extended follow-up data were retrospectively analyzed. This study was conducted according to the ethical principles of the Declaration of Helsinki and approved by the Ethics Committee of Daping Hospital and Army Medical Center of PLA (#2020-54). The clinicopathological characteristics of these patients are summarized in Table 1.

The neoadjuvant treatment regimen for the patients in this study was platinum combined with albumin-bound paclitaxel, of which 6 patients received cisplatin combined with albumin-bound paclitaxel, and the remaining 3 patients received carboplatin combined with albumin-bound paclitaxel due to renal inadequacy. Among them, 6 patients, 2 patients, and 1 patient received 2 cycles, 3 cycles, and 4 cycles of treatment, respectively. The assessment of clinical efficacy was performed by CT. The pathological T stage changes were the alteration value of the T stage compared to baseline (e.g., T3 to T1, 2). The pathological score was defined as 1 (0-33.3%), 2 (33.4-66.6%), and 3 (66.7-100%), respectively, according to the rate of the tumor response.

The therapeutic effect score is defined as the average of the pathological T stage changes and the pathological score after neoadjuvant chemotherapy. The average score of alteration value of the T stage compared to baseline (e.g., T3 to T1, 2) and tumor regression ratio by CT RECIST Version 1.1. The treatment outcome was stratified into good response (not less than 1.5) and poor response (less than 1.5) by the therapeutic effect score.

### 2.2. RNA Extraction, RNA-Seq Library Preparation, and Next Generation Sequencing

Total RNAs were extracted, purified, and eluted from Formalin-Fixed Paraffin-Embedded (FFPE) tissue sections by the truXTRAC™ FFPE RNA Kit (Covaris, Inc., Woburn, MA) and then quantified using the Quant-iT RNA HS Assay Kit (Thermo Fisher Scientific, Waltham, MA). RNA-Seq libraries were generated using the Ion AmpliSeq™ targeted sequencing system (Thermo Fisher Scientific, Waltham, MA). A total of 395 genes were quantified, including 10 housekeeping (HK) genes as controls. After reverse transcription into complementary DNA (cDNA), the barcode adapter was ligated to the partially digested amplicon. Equimolar libraries were pooled after purification and normalization and subjected to the Ion Chef™ system (Thermo Fisher Scientific) for enrichment and template preparation. Afterwards, 200 bp sequencing was performed to obtain mapped reads (approximately 2-3 million per sample) using the Ion Torrent S5 system (Thermo Fisher Scientific). Next-generation sequencing (NGS) data were subsequently analyzed using Ion Torrent Suite software version 5.2.0 (Thermo Fisher Scientific) for NGS read alignment, reference mapping, variant calling, and data management. NGS read quality control and quality assurance were conducted using standardized criteria [15]. Reads of HK genes were used for gene expression normalization. Finally, normalized reads per million (nRPM) were log2-transformed.

### 2.3. Selection of Genes

The content for the gene panel was selected to assess the tumor microenvironment and was based on literature, noting potentially predictive markers for drug response. The genes represented were carefully and extensively curated from multiple sources, which included over 200 peer-reviewed articles, input from experts at the Japan National Cancer Center, pharmaceutical companies, and public databases such as the Database for Annotation, Visualization, and Integrated Discovery (DAVID), and clinicaltrials.gov (the registry for clinical trials), as well as the Ion TorrentTM OncomineTM Knowledgebase, one of the world's largest collections of curated oncology data. This resulted in comprehensive coverage of targets associated with key genes expressed in the tumor microenvironment, as well as biomarkers involved in the immune response.

### 2.4. Hierarchal Clustering Heatmap and Gene Set Variation Analysis

Differentially expressed genes (DEGs) between the two groups were identified using the Limma package in R (version 3.4.1). Heatmap of the expression profiles of DEGs was constructed using the *z*-score normalization of each gene. In the heatmap, the rows represent genes, while the columns represent patients. The 395 immune transcripts from the RNA IO panel were annotated and classified, and the gene set variation analysis (GSVA) R package was used to analyze and cluster these DEGs according to the pathway and function.

### 2.5. Single-Sample Gene Set Enrichment Analysis (ssGSEA)

The tumor-infiltrating fraction of 28 immune cell subtypes in the tumor immune microenvironment [16] was calculated and quantified using the single sample gene set enrichment (ssGSEA) method (R library GSVA) [17]. These immune genes were found to be closely related to the tumor immune microenvironment.

### 2.6. Coexpression Module

Coexpression module was used to analyze the gene expression of 14 immune cell populations. The cell score was calculated as the average log2 normalized expression of each cell's marker genes. The total tumor-infiltrating lymphocyte (TIL) score was the average of all cell scores that have a correlation with PTRPC (CD45) greater than 0.6. The composite score excluded dendritic cells, Tregs, and mast cells. These scores are a measure of the abundance or depletion of each cell population relative to total tumor-infiltrating lymphocytes [18].

### 2.7. Innate Anti-PD-1 Resistance Signature (IPRES)

The coenrichment of 26 transcriptomic signatures, known as the innate anti-PD-1 resistance (IPRES) signature, indicates heightened mesenchymal transition, angiogenesis, hypoxia, and wound healing [19]. The ssGSEA method was used to calculate the level of IPRES in each sample to obtain the IPRES (enrichment) score.

### 2.8. Cytolytic Activity

Cytolytic activity (CYT) is based on the geometric mean of granzyme A (GZMA) and perforin 1 (PRF1) expression in transcripts per million (TPM), with an offset of 0.01. The expression of GZMA and PRF1 was dramatically upregulated during CD8+ T cell activation and clinical responses to anti-CTLA-4/PD-L1 immunotherapies [20].

### 2.9. T Cell-Inflamed Gene Expression Profiles

Penalized regression models were used as described previously to calculate T cell-inflamed gene expression profile (GEP) scores and derived a final set of 18 genes [21]. For the calculation of the T cell-inflamed GEP scores, the final regression coefficient values of the genes that have not been zeroed out by the penalty terms were used as the weights. The score was the sum of the weighted housekeeping normalized values of the 18 genes.

### 2.10. IHC Staining

Tumor specimens were fixed in 10% formaldehyde solution and embedded in paraffin. The paraffin-embedded tissues derived from clinical specimens of bladder cancer were sectioned at 4 *μ*m thickness and mounted on glass slides in sequence. After being baked at 60°C for 2 h, the sections were then deparaffinized in xylene, rehydrated through grade ethanol, and eliminated the endogenous peroxidase activity in 3% hydrogen peroxide. For antigen retrieval, the sections were submerged in citrate or EDTA buffer and boiled in the pressure cooker for 2 min. To block nonspecific background, goat serum (ZSGB-BIO, China) was applied. The sections were incubated overnight at 4°C with specific primary antibodies against LAG3, VEGF, and TNFSF14 and then incubated with horseradish peroxidase-conjugated anti-rabbit IgG secondary antibodies. After a brown stain was generated with DAB conjugated by horseradish peroxidase, slides were counterstained with hematoxylin, dehydrated, and covered with coverslips. All of the slides were assessed by two urological pathologists.

### 2.11. Enrichment Analysis

The enrichment analysis of the differentially expressed genes (DEGs) was performed by the ClusterProfiler package [22], which includes gene ontology (GO) and the Kyoto Encyclopedia of Genes and Genomes Pathway Database (KEGG). GO covers three domains, including molecular function, biological process, and cellular component.

### 2.12. Statistical Analysis

Statistical analyses were performed using R software (version 3.6.2, https://www.r-project.org/) and GraphPad Prism software (version 8). The Wilcoxon rank-sum test was used to assess hierarchical data, while the chi-square test or Fisher's exact test was used to assess binary data. A *P* value < 0.05 was considered statistically significant.

## 3. Results

### 3.1. Demographic and Clinical Characteristics

A total of 9 male patients were included in the analysis of this study. Patient characteristics and treatment responses are shown in Table 1. Among them, 5 patients had matched specimens before and after neoadjuvant therapy, while the other 4 patients had only specimens before (2 cases) or after neoadjuvant therapy (2 cases). Six of the nine patients had smoking history. The tumor stage of patients was preoperative T2 stage for 6 patients and T3 stage for 3 patients. The American Joint Committee on Cancer (AJCC) stages of the patients were stage II for 7 patients and stage IIIA for 2 patients. After receiving neoadjuvant therapy, 1 patient, 5 patients, and 3 patients archived complete remission, partial remission, and stable disease, respectively. After followed up until December 2020, 2 patients developed lung metastasis or pelvic metastasis.

### 3.2. Effect of Neoadjuvant Chemotherapy on the Transcriptomic Signatures of Bladder Cancer in the Immune Microenvironment

**Figure 1:**
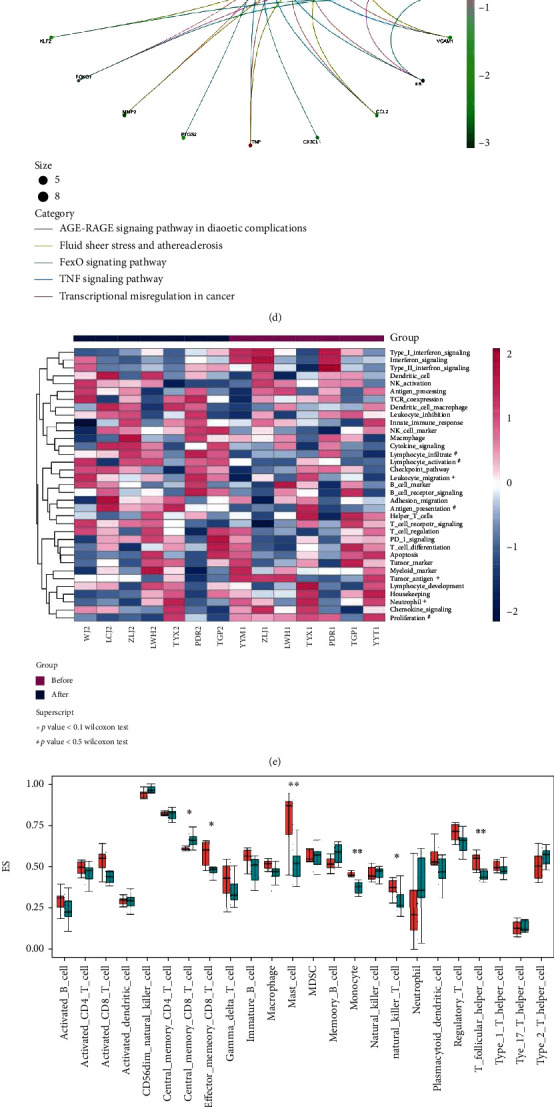
Transcriptomic signatures of bladder cancer in the immune microenvironment before and after neoadjuvant chemotherapy. (a) Heatmap showing the differentially expressed genes in bladder cancer tissue samples. (b) Bar plot showing the differentially expressed genes with significant fold changes. (c) Gene ontology (GO) analysis. (d) Kyoto Encyclopedia of Genes and Genomes (KEGG) analysis. (e) Hierarchical clustering heatmap showing the gene set variation analysis (GSVA) of gene signatures. (f) Bar plot showing immune infiltration genes analyzed by single-sample gene set enrichment analysis (ssGSEA). (g) Hierarchical clustering heatmap for innate anti-PD-1 resistance signature (IPRES). (h) Bar plot showing the score and fold change of T cell-inflamed gene expression profile (GEP). *P* value was calculated by the Wilcoxon rank-sum test or described in the panel. Differences were found to be statistically significant at ^∗^*P* < 0.05 and ^∗∗^*P* < 0.01.

In order to characterize the immune microenvironment of urinary bladder carcinoma, an RNA immune oncology (IO) profiling was used to simultaneously measure the response of 395 immune-related genes (Supplementary Material 1) in a single reaction. After analyzing and comparing the gene expression profiles of tissue samples from 9 patients before and after neoadjuvant chemotherapy. A total of 43 genes were significantly upregulated, while 10 genes were significantly downregulated (*P* < 0.05, Figures 1(a) and 1(b)). As shown in Figure 1(b), the top 6 upregulated genes were neural cell adhesion molecule 1 (NCAM1), interleukin 6 (IL-6), early growth response 3 (EGR3), C-C motif chemokine ligand 21 (CCL21), matrix metallopeptidase 2 (MMP2), and early growth response 2 (EGR2). The top 6 downregulated genes were maternal embryonic leucine zipper kinase (MELK), B melanoma antigen (BAGE), major histocompatibility complex class I C (HLA-C), KIAA0101, MAD2 mitotic arrest deficient-like 1 yeast (MAD2L1), and topoisomerase DNA II alpha (TOP2A). These upregulated genes were functionally involved in 19 pathways, such as lymphocyte infiltration (10 genes), cytokine signaling (4 genes), checkpoint pathway (4 genes), TCR coexpression (3 genes), innate immune response (2 genes), and antigen presentation (2 genes) (Table 2 and Supplemental Figures 1a–b). In contrast, these downregulated genes were functionally involved in proliferation, neutrophil, checkpoint pathway, antigen processing, and tumor antigen.  Figure 1(c) represents the top 26 most representative GO terms from the GO enrichment analysis.  Figure 1(d) shows the top 5 enriched KEGG terms from KEGG pathway enrichment analysis. GSVA analysis showed that 8 gene sets were significantly changed after neoadjuvant chemotherapy. The enrichment score increased in the pathway of lymphocyte infiltration, lymphocyte activation, leukocyte migration, and antigen presentation but decreased in the pathway of type I interferon signaling, tumor antigen, neutrophil, and proliferation (Figure 1(e)). The infiltration levels of several immune cells changed significantly after neoadjuvant chemotherapy. As shown in Figure 1(f), effector memory CD8 T cell, mast cell, monocyte, natural killer T (NKT) cell, and T follicular helper (Tfh) cell were significantly increased after neoadjuvant chemotherapy, while central memory CD8 T cell decreased. In addition, the results of IPRSE, GEP, and CYT analysis showed that neoadjuvant chemotherapy significantly increased the response to immune checkpoint blockade (Figures 1(g) and 1(h) and Supplemental Figure 1d–f).

### 3.3. Identification of Differentially Expressed Immune-Related Gene Related to Efficacy before Neoadjuvant Chemotherapy

**Figure 2:**
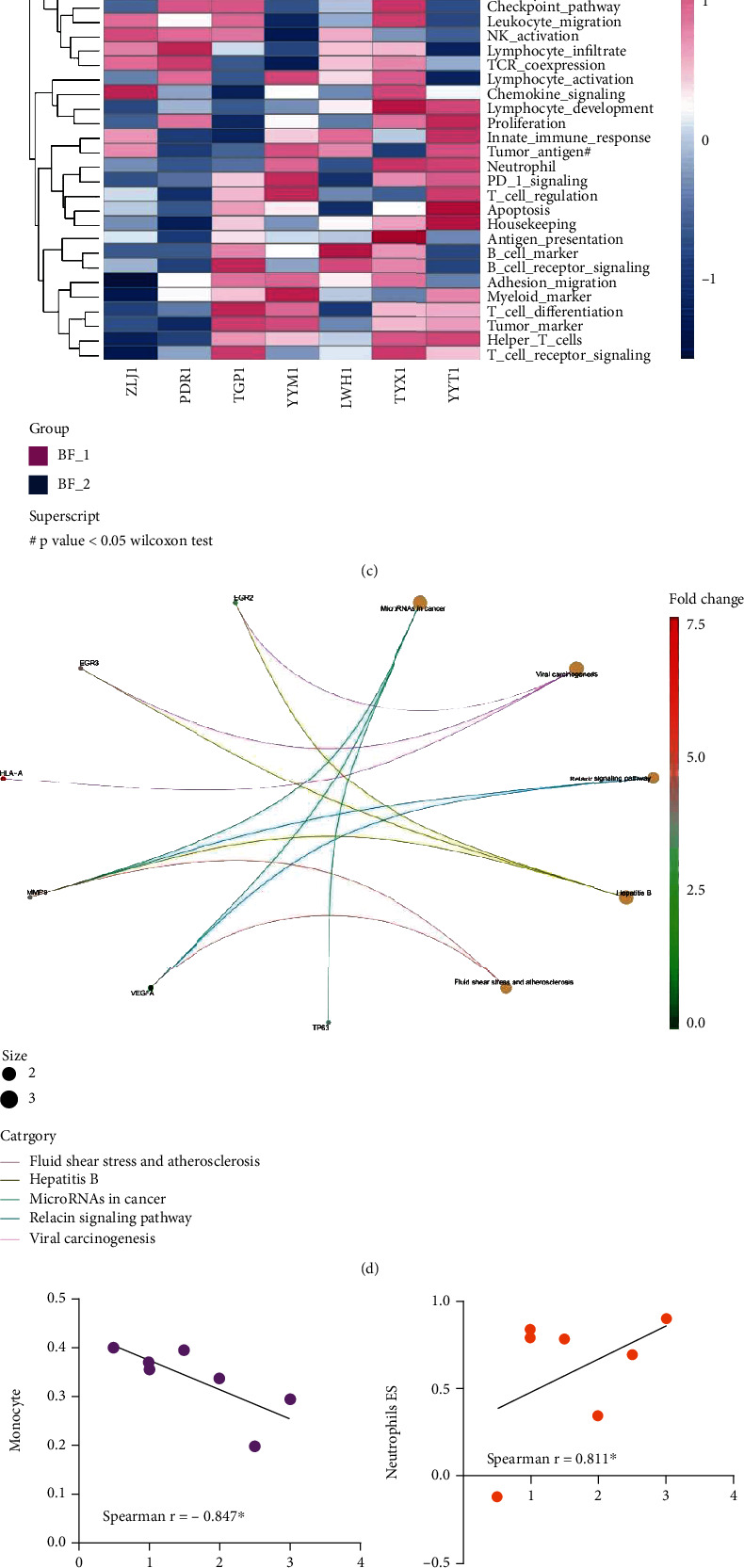
Identification of differentially expressed immune-related genes related to efficacy before neoadjuvant chemotherapy. Seven cases of bladder cancer tissues before neoadjuvant chemotherapy were divided into a favorable prognosis group (*n* = 4) and a poor prognosis group (*n* = 3) according to the final curative effect. (a) Heatmap showing the differentially expressed genes. (b) Bar plot showing the differentially expressed genes with significant fold change. (c) Hierarchical clustering heatmap showing the gene set variation analysis (GSVA) of gene signatures. (d) Chord diagram showing the top 5 enriched KEGG terms for 6 differentially expressed genes. (e) The Spearman correlation analysis between therapeutic effect score and monocyte. (f) The Spearman correlation analysis between therapeutic effect score and neutrophil. (g) The Spearman correlation analysis between pathological score and Th1 cell. (h) The pathway interaction network showing the relationship with pathological score and therapeutic effect score by IPRSE analysis. *P* values in the (a) and (b) subfigures were calculated by the Wilcoxon rank-sum test, while *P* values in the (e–h) subfigures were calculated by Spearman correlation analysis. Differences were found to be statistically significant at ^∗^*P* < 0.05.

**Figure 3:**
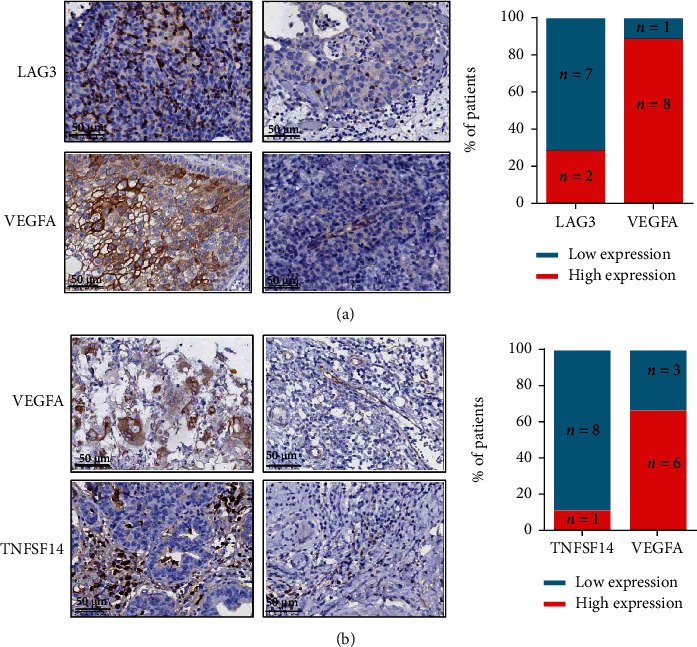
IHC of differentially expressed immune-related genes before and after neoadjuvant chemotherapy. The tumor tissues from patients before neoadjuvant chemotherapy were stained for (a) LAG3 and VEGFA and, after neoadjuvant chemotherapy, were stained for (b) VEGFA and TNFSF14. Magnifications: ×400.

In order to identify the DEGs that are beneficial to neoadjuvant chemotherapy, the cancer tissues of patients before treatment were divided into a favorable prognosis group and a poor prognosis group according to the final treatment effect. A total of 3 genes had significantly higher expression in the favorable prognosis group, including GATA3, VEGFA, and IKZF2 (Figures 2(a) and 2(b)). In contrast, a total of 12 genes had significantly lower expression in the favorable prognosis group, including EGR2, EGR3, HLA-A, TP63, HERC6, HLA-F-AS1, CXCL10, LAG3, IFIT1, IFITM1, MMP9, and TAGAP. Among them, IKZF2, GATA3, and VEGFA (Figure 3(a)) genes were functionally involved in the pathway of lymphocyte development, helper T cells, and chemokine signaling, respectively (Table 3). The 12 downregulated genes were functionally involved in 8 pathways, including tumor maker (TP63, MMP9, and EGR3 genes) and antigen processing (HLA-F-AS1 and HLA-A genes). KEGG pathway enrichment analysis showed that EGR2, EGR3, HLA-A, MMP9, VEGFA, and TP65 genes are involved in microRNAs in cancer, viral carcinogenesis, relaxin signaling pathway, hepatitis B, fluid shear stress, and atherosclerosis (Figure 2(c)), suggesting that microRNAs, viruses, etc., may affect the efficacy of neoadjuvant chemotherapy. Heatmap of GSVA analysis showed that low leukocyte inhibition and high tumor antigen may affect the efficacy of neoadjuvant chemotherapy (Figure 2(d)). Although there were no significant changes in immune cell infiltration between the favorable prognosis group and the poor prognosis group, we found that monocyte and neutrophil were related to therapeutic effect score (Figures 2(e) and 2(f)), while type I T helper cell was related to pathological score (Figure 2(g)). In addition, IPRSE analysis was further conducted to explore the relationship with pathological scores, and the results showed that the efficacy of neoadjuvant chemotherapy in bladder cancer patients was positively correlated with the response to immune checkpoint blockade (Figures 2(h) and 3(a)).

### 3.4. Identification of Differentially Expressed Immune-Related Genes Related to Efficacy after Neoadjuvant Therapy

**Figure 4:**
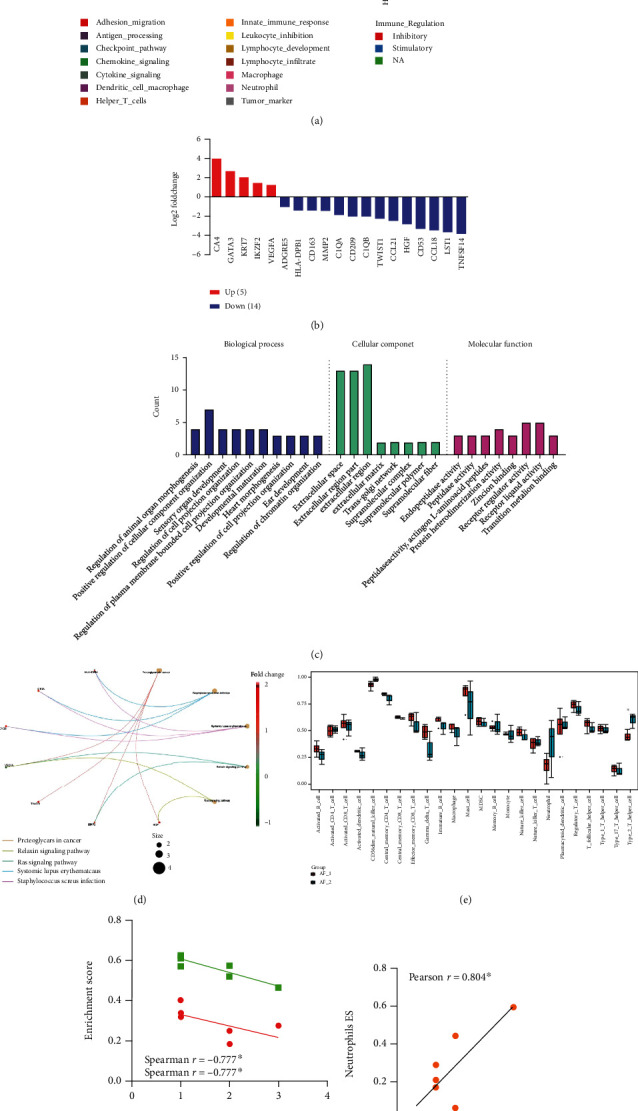
Identification of differentially expressed immune-related genes related to efficacy after neoadjuvant therapy. Seven cases of bladder cancer tissues after neoadjuvant chemotherapy were divided into the favorable prognosis group (*n* = 3) and the poor prognosis group (*n* = 4) according to the final curative effect. (a) Heatmap showing the differentially expressed genes with significant fold change. (b) Bar plot showing the differentially expressed genes with significant fold change. (c) Gene ontology (GO) analysis. (d) Kyoto Encyclopedia of Genes and Genomes (KEGG) analysis. (e) Bar plot showing immune infiltration genes analyzed by single-sample gene set enrichment analysis (ssGSEA). (f) The Spearman correlation analysis between pathological score and activated/immature B cell. (g) The Pearson correlation analysis between therapeutic effect score and neutrophil. *P* values in the (a), (b), and (e) subfigures were calculated by the Wilcoxon rank-sum test (two-tailed). The *P* values in the (f) and (g) subfigure were calculated by Spearman correlation analysis and Pearson correlation analysis, respectively. Differences were found to be statistically significant at ^∗^*P* < 0.05.

The postoperative cancer tissues of patients were divided into a favorable prognosis group and a poor prognosis group according to the final curative effect. A total of 19 DEGs were identified (Figures 4(a) and 4(b)). Compared with the poor prognosis group, the expression of 5 genes (CA4, GATA3, KRT7, and VEGFA (Figure 3(b))) in the favorite prognosis group was significantly upregulated, and the expression of 14 genes was significantly downregulated (Figures 4(a) and 4(b), Table 4, and Figure 3(b)). In addition, KEGG pathways and GO enrichment analysis of these DEGs were conducted to reveal the enrichment status between the two groups (Figures 4(c) and 4(d)). These DEGs enriched in type 2 T helper cell in the favorite prognosis group was significantly higher than that in the poor prognosis group (Figure 4(e)). In addition, activated B cell and immature B cell were negatively correlated with pathological score (Figure 4(f)), while neutrophil was positively correlated with therapeutic effect score (Figure 4(g)).

### 3.5. The Expression of Immune-Related Genes upon before and after Neoadjuvant Chemotherapy in Bladder Cancer Tissue

We analyzed the expression of genes related to the checkpoint pathway in the sequencing results and found that the patients before neoadjuvant chemotherapy 77.8% (7/9) had low expression of LAG3, and 88.9% (8/9) patients had high expression of VEGFA. Of the patients after neoadjuvant chemotherapy, 88.9% (8/9) had low expression of TNFSF14, and 66.7% (6/9) patients had high expression of VEGFA. These tissue expression results are consistent with our sequencing results.

## 4. Discussion

The results of this study proved the relationship between tumor immune microenvironment, immune checkpoint blockade response, and neoadjuvant chemotherapy in patients with bladder cancer. The results not only reveal the status of TILs in the tumor microenvironment of bladder cancer but also found that neoadjuvant chemotherapy can regulate the expression of a series of genes involved in the activation of antitumor immune response, tumor proliferation pathway, and immune checkpoint blockade response. In addition, this study identified several candidate genes involved in the prognosis of the efficacy of neoadjuvant chemotherapy and their possible molecular mechanisms. We also reported the differences in genes, pathways, and TILs between good prognosis tissues and poor prognosis tissues after neoadjuvant chemotherapy. Therefore, the results of this study at least partially explain the impact of neoadjuvant chemotherapy on the tumor immune microenvironment of bladder cancer patients and shed light on certain groups of bladder cancer patients who may benefit from neoadjuvant chemotherapy.

Chemotherapeutic drugs are a kind of cytotoxic drugs, which mainly kill tumor cells by affecting biological events such as DNA replication, transcription, and microtubule stability of tumor cells. In addition to tumor cells, other rapidly dividing normal cells will be also killed by chemotherapy drugs. In the same way, T cells and other types of immune cells will also be targeted by these chemotherapeutic drugs, simply because of their rapid proliferation characteristics. Previous studies have shown that the chemotherapeutic drugs have a great influence on the immunosuppression and antitumor function of immune cells [23–25]. Several studies also demonstrate that the reduction of neutrophils is one of the side effects of the toxicity of chemotherapeutic drugs [26, 27]. Consistently, the results of this study showed that after neoadjuvant chemotherapy, genes associated with cell proliferation (CCNB2, TOP2A, MAD2L1, KIAA0101, and MELK) and neutrophil (KREMEN1 and DGAT2) decreased significantly. In contrast, after neoadjuvant chemotherapy, genes associated with antitumor function of immune cells increased, such as lymphocyte (infiltrate, activation, and development), innate immune response, NK activation, NK cell marker, and dendritic cell. Genes related to effector memory CD8 T cell, mast cell, monocyte, NKT cell, and Tfh cell increased significantly. Several studies have found that high infiltration of CD8+ T cells is associated with improved survival in patients with bladder cancer [28–31]. Tfh cells are a specific subset of CD4+ T cells located in B cell follicles, which can provide cytokines, regulate the interaction of B cells and T cells in germplasm centers (GC), facilitate B cell differentiation into memory B cells or plasma cells, and promote the generation of high-affinity antibodies [32–34]. Recent studies also found that colorectal cancer or breast cancer patients with higher Tfh cells infiltration have better survival [35, 36], and mast cell and NKT are also considered to be good prognostic biomarkers for many types of cancer [37–40]. Our results revealed that the genes associated with lymphocyte development (IKZF2), helper T cells (GATA3), and chemokine signaling (VEGFA) were all increased significantly in favorable prognosis patients before and after neoadjuvant chemotherapy. The neutrophil was correlated with the prognosis between the two groups before and after neoadjuvant chemotherapy. In addition, significant changes in type 2 T helper cell infiltration were observed after neoadjuvant chemotherapy between the two groups. The findings may identify several candidate genes to predict the prognosis of the efficacy of neoadjuvant chemotherapy.

The prognosis and the response to therapy in the bladder cancer are also tightly associated with immune activation status [41]. Tregs and MDSCs can reflect the immune-exhausted status in the TME [42, 43]. In this study, we found that genes associated with T cell regulation and myeloid markers also increased after neoadjuvant chemotherapy. Although Treg is associated with poor prognosis of breast cancer, oral squamous cell carcinoma, non-small-cell lung cancer, and hepatocellular carcinoma [44–47], a study by Winerdal et al. demonstrated that the high expression of Treg is associated with good prognosis of patients with bladder cancer [48]. One of the mechanisms by which tumor cells reshape the immunosuppressive microenvironment is to promote the infiltration of immunosuppressive Treg cells and myeloid-derived suppressor cells into tumor tissues by releasing cytokine. Our results did not definitely discriminate the potential alteration of immune microenvironment activation or suppression status after neoadjuvant therapy, and further studies were needed. Therefore, the final immune response caused by chemotherapeutic drugs will depend on the final balance between the suppression and activation of the tumor immune microenvironment.

Chemotherapeutic drugs can affect the immunogenicity of tumor cells through complex mechanisms, including affecting antigen release and inducing immunogenic cell death [49]. In addition to acting directly on immune cells, the effect of chemotherapeutic drugs on immune cells is also through the indirect effect on tumor cells. The killing effect of immune cells on tumor cells is a complex process, which involves the interaction between multiple immune cells and targeted tumor cells. Briefly, antigen-presenting cells (e.g., macrophage or dendritic cells) can recognize and process tumor-specific antigens and present them to effector cells, such as T cells. Afterwards, T cells can recognize and kill these tumor cells that contain the same tumor-specific antigens. Thus, the recognition of tumor antigen by antigen-presenting cells is an important process. It is known that chemotherapy against tumor cells requires the participation of the immune system. Once the tumor immunogenic cell death is induced, the ratio of cytotoxic T cells to Treg in the tumors increases, and the patient has a good prognosis [50, 51]. The process of immunogenic cell death includes ATP release, calreticulin membrane translocation, and the release of high mobility group box 1 (HMGB1). Subsequently, innate immune cells are activated to recognize the antigens released from these dead tumor cells and then cross-present them to effector T cells, ultimately enhancing the antitumor immune response.

In this study, we found that after neoadjuvant chemotherapy, genes associated with antigen presentation, TCR coexpression, innate immune response, NK activation, NK cell marker, DC lymphocyte (infiltrate, activation, and development), cytokine signaling, effector memory CD8 T cell, mast cell, monocyte, NKT cell, and Tfh cell were significantly increased. These findings suggest that neoadjuvant chemotherapy for patients with bladder cancer may affect a series of genes related to immune checkpoint blockade.

Cell senescence may also occur in damaged cells. When cell damage reaches a certain limit and cannot be repaired, the cells will stop proliferating and present a senescence phenotype, which is an important protective mechanism to prevent the proliferation of the damaged cells [52]. Although the proliferation of senescent cells has stopped, the gene transcription of the cells is still active, especially the expression and secretion of inflammatory cytokines, such as IL-6, CCL2, and CCL16. This phenomenon is known as senescent-associated secretory phenotype (SASP) [53]. Studies have shown that DNA damage caused by cisplatin treatment can trigger tumor cell senescence and further induce SASP and the release of inflammatory factors [54]. CCL2 can recruit the macrophage to infiltrate and engulf these senescent tumor cells. In addition, CXCL16 can recruit CD4+ T cells and NKT cells to kill these senescent aging tumor cells [55, 56]. In this study, we found that neoadjuvant chemotherapy affected genes related to lymphocyte infiltrate and cytokine signaling, including CCL21, CCL2, CXCR4, and IL-6. These findings suggest that neoadjuvant chemotherapy may induce SASP.

With the recent success of immune checkpoint blockade therapies in inducing durable control of multiple tumors [57, 58], predicting whether tumors will be resistant to therapy has become critical. At present, models for predicting the curative effect of immune checkpoint blockade had been successfully developed, including IPRSE, GEP, and CYT. Our results suggest that neoadjuvant chemotherapy may have the potential to combine immune checkpoint blockade therapy to treat patients with bladder cancer. It is likely that neoadjuvant chemotherapy may transform noninflammatory tumors (cold tumors) into tumors enriched with cytotoxic cells (hot tumors). As for specific clinical treatment protocol, there is currently no relevant study or report at present, and it warrants further in-depth research in the future.

This study has some limitations. First, our results were based on the analysis of 9 patients with bladder cancer. Although the sample size is not large, we still found several genes related to immunosuppressive microenvironment and immune checkpoint blockade. The sample should be expanded in the future to identify more other genes. Second, the 395 analyzed genes in this study were from the RNA immune-oncology profiling panel, which mainly include immune genes and 10 housekeeping genes. Therefore, it is still unclear in terms of transcriptomics, genomics, and proteinomics. It warrants further investigation.

## 5. Conclusion

Our study revealed that neoadjuvant chemotherapy has active antitumor effects, improves the suppressive tumor immune microenvironment, and finally increases the sensitivity of bladder cancer patients to immune checkpoint blockade.

## Figures and Tables

**Table 1:** Characteristics of patients.

Characteristic	Category	All case *N* (%)	Sequencing cases *N* (%)
Age	Median	66 (50-80)	66 (50-77)

Sex	Female	3	0 (0)
	Male	21	9 (100)

Tumor status	T2	17	6
	T3	7	3

Nodal status	Positive	2	0
	Negative	22	9

AJCC stage	II	16	7
	IIIA	8	2

Neoadjuvant chemotherapy	Albumin paclitaxel combined with cisplatin	14	6
	Albumin paclitaxel combined with carboplatin	10	3

Frequency of chemotherapy	2 cycles	19	6
	3 cycles	1	2
	4 cycles	4	1

ORR	CR	2	1
	PR	16	4
	SD	5	4
	PD	1	0

**Table 2:** Molecular changes after neoadjuvant chemotherapy.

	Pathway	Gene count	Gene
Upregulated	Lymphocyte infiltrate	10	CCL21, CCL2, CXCR4, PTPRC, IL-10RA, FYB, CD52, SRGN, TYROBP, TNFAIP8
	Tumor marker	4	EGR3, MMP2, PTGS2, ZEB1
	Cytokine signaling	4	IL-6, IL-4, CSF1R, CSF2RB
	Checkpoint pathway	4	CD69, PDCD1LG2, CD28, ENTPD1
	TCR coexpression	3	ITK, CD3E, IL-7R
	Adhesion_migration	3	NCAM1, ITGA1, ADGRE5
	Innate_immune_response	2	LYZ, AXL
	Antigen_presentation	2	CD1C, CD83
	Type_II_interferon_signaling	1	CX3CL1
	T_cell_regulation	1	KLF2
	T_cell_differentiation	1	EGR2
	PD_1_signaling	1	FOXO1
	NK_cell_marker	1	NCR3
	NK_activation	1	KLRG1
	Myeloid_marker	1	MPO
	Lymphocyte_development	1	IKZF1
	Lymphocyte_activation	1	SH2D1A
	Leukocyte_migration	1	VCAM1
	Dendritic_cell	1	NRP1

Downregulated	Proliferation	5	CCNB2, TOP2A, MAD2L1, KIAA0101, MELK
	Neutrophil	2	KREMEN1, DGAT2
	Checkpoint_pathway	1	TNF
	Antigen_processing	1	HLA-C
	Tumor_antigen	1	BAGE

**Table 3:** Differences of a gene in patients with different curative effects before treatment.

	Pathway	Gene count	Gene
Upregulated	Helper_T_cells	1	GATA3
	Lymphocyte_development	1	IKZF2
	Chemokine_signaling	1	VEGFA

Downregulated	Tumor_marker	3	TP63, MMP9, EGR3
	Antigen_processing	2	HLA-F-AS1, HLA-A
	Type_I_interferon_signaling	2	IFITM1, IFIT1
	Dendritic_cell	1	HERC6
	Lymphocyte_infiltrate	1	TAGAP
	T_cell_differentiation	1	EGR2
	Checkpoint_pathway	1	LAG3
	Type_II_interferon_signaling	1	CXCL10

**Table 4:** Differences of a gene in patients with different curative effects after neoadjuvant chemotherapy.

	Pathway	Gene count	Gene
Upregulated	Neutrophil	1	CA4
	Helper_T_cells	1	GATA3
	Tumor_marker	1	KRT7
	Lymphocyte_development	1	IKZF2
	Chemokine_signaling	1	VEGFA

Downregulated	Adhesion_migration	2	ADGRE5, CD53
	Tumor_marker	2	MMP2, TWIST1
	Innate_immune_response	2	C1QA, C1QB
	Lymphocyte_infiltrate	2	CCL21, CCL18
	Antigen_processing	1	HLA-DPB1
	Macrophage	1	CD163
	Dendritic_cell_macrophage	1	CD209
	Cytokine_signaling	1	HGF
	Leukocyte_inhibition	1	LST1
	Checkpoint_pathway	1	TNFSF14

## Data Availability

The datasets used and analyzed during the current study are available from the corresponding author on reasonable request.

## Supplementary Materials

Supplementary 1 Supplemental Figure 1: (a) circular diagram of upregulated pathways of 9 patients before and after neoadjuvant therapy. (b) Circular diagram of downregulated pathways of 9 patients before and after neoadjuvant therapy. (c) TIL score of 14 immune cell subtypes of 9 patients before and after neoadjuvant therapy. TIL scores were calculated by coexpression module. The Wilcoxon rank-sum test was used to compare the two paired groups. Differences were found to be statistically significant at ^∗^*P* < 0.05 (two-tailed test). (d) Bar plot showing the log2 fold change difference of two CYT-related genes (GZMA and PRF1) and CYT value before and after neoadjuvant therapy in 9 patients. (e) Bar plot showing the delta enrichment score of IPRES of 5 paired tissues. Differences were found to be statistically significant at ^∗^*P* < 0.05 and ^∗∗^*P* < 0.01 (two-tailed *t*-test). (f) Network diagram showing the relationship between delta enrichment score of pathways in IPRSE analysis, pathological score, and therapeutic effect score. The association between groups was examined using Spearman correlation analysis. A ^∗^*P* < 0.05 was considered statistically significant.

Supplementary 2 The gene list of each signature used in this study.
