# Supplementary material for: Neoadjuvant Chemotherapy Improves the Immunosuppressive Microenvironment of Bladder Cancer and Increases the Sensitivity to Immune Checkpoint Blockade
Source: J Immunol Res. 2022 Jul 21;2022:9962397. doi: 10.1155/2022/9962397 (PMC9338739; doi:10.1155/2022/9962397)
Supplement: Supplementary 1 — Supplemental Figure 1: (a) circular diagram of upregulated pathways of 9 patients before and after neoadjuvant therapy. (b) Circular diagram of downregulated pathways of 9 patients before and after neoadjuvant therapy. (c) TIL score of 14 immune cell subtypes of 9 patients before and after neoadjuvant therapy. TIL scores were calculated by coexpression module. The Wilcoxon rank-sum test was used to compare the two paired groups. Differences were found to be statistically significant at ∗P < 0.05 (two-tailed test). (d) Bar plot showing the log2 fold change difference of two CYT-related genes (GZMA and PRF1) and CYT value before and after neoadjuvant therapy in 9 patients. (e) Bar plot showing the delta enrichment score of IPRES of 5 paired tissues. Differences were found to be statistically significant at ∗P < 0.05 and ∗∗P < 0.01 (two-tailed t-test). (f) Network diagram showing the relationship between delta enrichment score of pathways in IPRSE analysis, pathological score, and therapeutic effect score. The association between groups was examined using Spearman correlation analysis. A ∗P < 0.05 was considered statistically significant. [file 9962397.f1.docx]

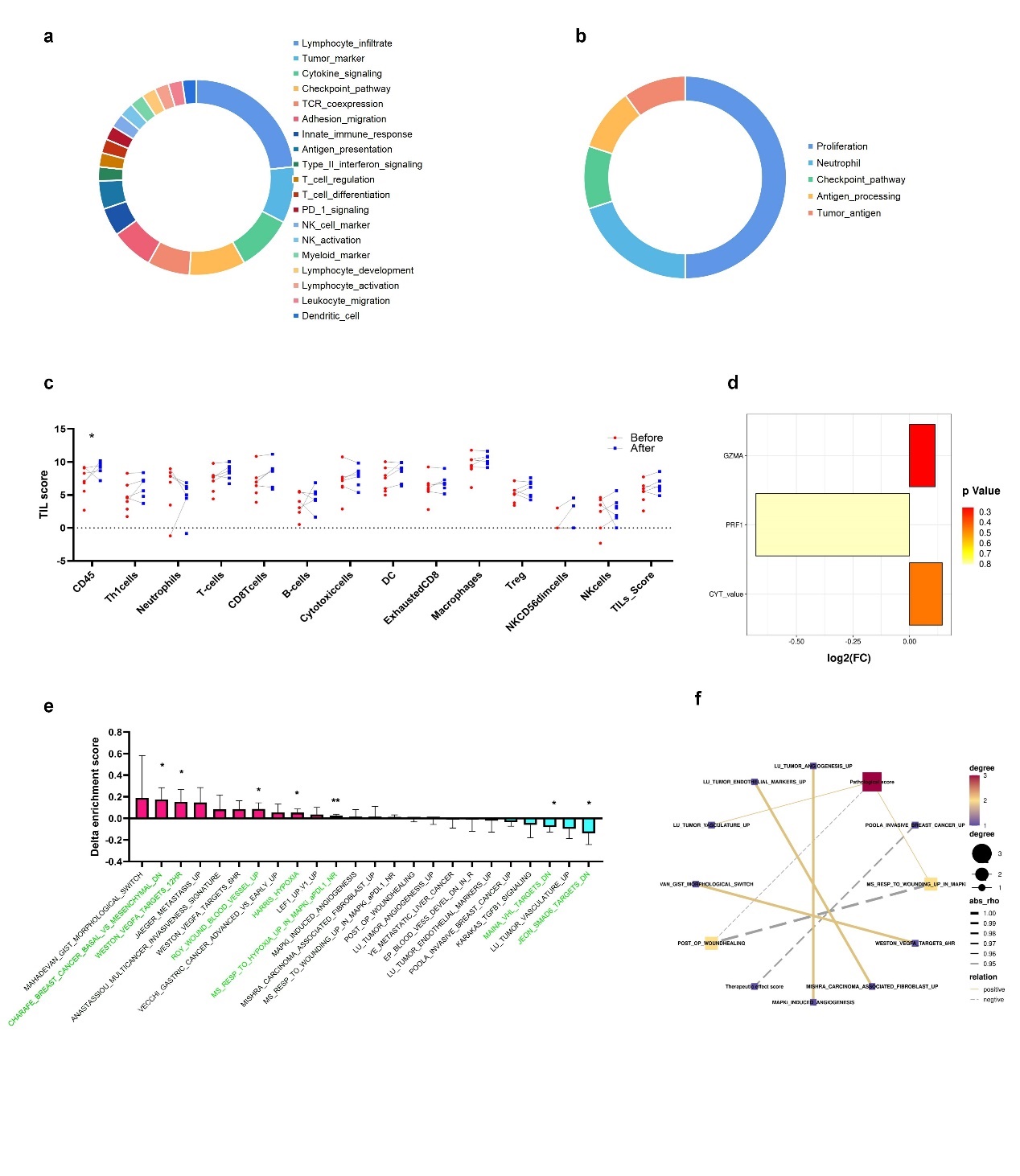


**Supplemental Figure 1 legend**

**a.** Circular diagram of up-regulated pathways of 9 patients before and after neoadjuvant therapy. **b**. Circular diagram of down-regulated pathways of 9 patients before and after neoadjuvant therapy. **c**. TIL score of 14 immune cell subtypes of 9 patients before and after neoadjuvant therapy. TIL scores were calculated by Co-expression module. The Wilcoxon rank-sum test was used to compare the two paired groups. Differences were found to be statistically significant at *P<0.05 (two-tailed test). **d**. Bar plot showing the log2 fold change difference of two CYT-related genes (GZMA and PRF1) and CYT value before and after neoadjuvant therapy in 9 patients. **e**. Bar plot showing the delta enrichment score of IPRES of 5 paired tissues. Differences were found to be statistically significant at *P<0.05 and **P<0.01 (two-tailed t-test). f. Network diagram showing the relationship between delta enrichment score of pathways in IPRSE analysis, pathological score and therapeutic effect score. The association between groups was examined using Spearman correlation analysis. A *P<0.05 was considered statistically significant.
