# Supplementary material for: Neoadjuvant Chemotherapy Improves the Immunosuppressive Microenvironment of Bladder Cancer and Increases the Sensitivity to Immune Checkpoint Blockade
Source: J Immunol Res. 2022 Jul 21;2022:9962397. doi: 10.1155/2022/9962397 (PMC9338739; doi:10.1155/2022/9962397)
Supplement: Supplementary 2 — The gene list of each signature used in this study. [file 9962397.f2.pdf]

NCBI Accession ID	Gene Symbol	Gene name	Gene function
NM_078481	ADGRE5	adhesion G protein-coupled receptor	Adhesion,migration
NM_006566	CD226	CD226 molecule	
NM_001775	CD38	CD38 molecule	
NM_000610	CD44	CD44 molecule Indian blood group	
NM_001777	CD47	CD47 molecule	
NM_001040033	CD53	CD53 molecule	
NM_000875	IGF1R	insulin like growth factor 1 receptor	
NM_181501	ITGA1	integrin subunit alpha 1	
NM_002208	ITGAE	integrin subunit alpha E	
NM_002211	ITGB1	integrin subunit beta 1	
NM_130760	MADCAM1	mucosal vascular addressin cell	
NM_181351	NCAM1	neural cell adhesion molecule 1	
NM_001042724	NECTIN2	nectin cell adhesion molecule 2	
NM_000442	PECAM1	platelet and endothelial cell adhesion	
NM_001765	CD1C	CD1c molecule	Antigen presentation
NM_001766	CD1D	CD1d molecule	
NM_004233	CD83	CD83 molecule	
NM_001025159	CD74	CD74 molecule	Antigen processing
NM_002116	HLA-A	major histocompatibility complex class I	
NM_005514	HLA-B	major histocompatibility complex class	
NM_002117	HLA-C	major histocompatibility complex class	
NM_006120	HLA-DMA	major histocompatibility complex class	
NM_002118	HLA-DMB	major histocompatibility complex class	
NM_002119	HLA-DOA	major histocompatibility complex class	
NM_002120	HLA-DOB	major histocompatibility complex class	
NM_033554	HLA-DPA1	major histocompatibility complex class	
NM_002121	HLA-DPB1	major histocompatibility complex class	
NM_002122	HLA-DQA1	major histocompatibility complex class	
NM_020056	HLA-DQA2	major histocompatibility complex class	
NM_001198858	HLA-DQB2	major histocompatibility complex class	
NM_019111	HLA-DRA	major histocompatibility complex class	
NM_002124	HLA-DRB1	major histocompatibility complex class	
NM_005516	HLA-E	major histocompatibility complex class	
NM_001098479	HLA-F	major histocompatibility complex class I	
NR_026972	HLA-F-AS1	HLA-F antisense RNA 1	
NM_002127	HLA-G	major histocompatibility complex class	
NM_000633	BCL2	B-cell CLL lymphoma 2	Apoptosis
NM_138621	BCL2L11	BCL2 like 11	
NM_052850	GADD45GIP1	GADD45G interacting protein 1	
NM_003810	TNFSF10	tumor necrosis factor superfamily	
NM_001178098	CD19	CD19 molecule	
NM_001771	CD22	CD22 molecule	
NM_000566	FCGR1A	Fc fragment of IgG receptor Ia	
NM_004001	FCGR2B	Fc fragment of IgG receptor IIb	

NM_001184866	FCRLA	Fc receptor like A	B cell marker
NM_144646	JCHAIN	joining chain of multimeric IgA and IgM	
NM_006181	NTN3	netrin 3	
NM_006235	POU2AF1	POU class 2 associating factor 1	
NM_003930	SKAP2	src kinase associated phosphoprotein 2	
NM_001192	TNFRSF17	tumor necrosis factor receptor	
NM_006573	TNFSF13B	tumor necrosis factor superfamily	
NM_001039933	CD79B	CD79b molecule	B cell receptor signaling
NM_001783	CD79A	CD79a molecule	
NM_000043	FAS	Fas cell surface death receptor	
NM_000675	ADORA2A	adenosine A2a receptor	Checkpoint pathway
NM_181780	BTLA	B and T lymphocyte associated	
NM_022153	C10orf54	chromosome 10 open reading frame 54	
NM_007053	CD160	CD160 molecule	
NM_001166663	CD244	CD244 molecule	
NM_014143	CD274	CD274 molecule	
NM_001024736	CD276	CD276 molecule	
NM_006139	CD28	CD28 molecule	
NM_001778	CD48	CD48 molecule	
NM_001781	CD69	CD69 molecule	
NM_005191	CD80	CD80 molecule	
NM_175862	CD86	CD86 molecule	
NM_001712	CEACAM1	carcinoembryonic antigen related cell	
NM_001098175	ENTPD1	ectonucleoside triphosphate	
NM_005442	EOMES	eomesodermin	
NM_032782	HAVCR2	hepatitis A virus cellular receptor 2	
NM_012092	ICOS	inducible T-cell costimulator	
NM_015259	ICOSLG	inducible T-cell costimulator ligand	
NM_194294	IDO2	indoleamine 23-dioxygenase 2	
NM_002526	NT5E	5-nucleotidase ecto	
NM_025239	PDCD1LG2	programmed cell death 1 ligand 2	
NM_006505	PVR	poliovirus receptor	
NM_005651	TDO2	tryptophan 23-dioxygenase	
NM_000660	TGFB1	transforming growth factor beta 1	
NM_000594	TNF	tumor necrosis factor	
NM_003820	TNFRSF14	tumor necrosis factor receptor	
NM_003807	TNFSF14	tumor necrosis factor superfamily	
NM_005092	TNFSF18	tumor necrosis factor superfamily	
NM_003326	TNFSF4	tumor necrosis factor superfamily	
NM_024626	VTCN1	V-set domain containing T cell	
NM_002987	CCL17	C-C motif chemokine ligand 17	Chemokine signaling
NM_004591	CCL20	C-C motif chemokine ligand 20	
NM_002990	CCL22	C-C motif chemokine ligand 22	
NM_005508	CCR4	C-C motif chemokine receptor 4	
NM_004367	CCR6	C-C motif chemokine receptor 6	
NM_001511	CXCL1	C-X-C motif chemokine ligand 1	

NM_001557	CXCR2	C-X-C motif chemokine receptor 2	
NM_001504	CXCR3	C-X-C motif chemokine receptor 3	
NM_000265	NCF1	neutrophil cytosolic factor 1	
NM_001171623	VEGFA	vascular endothelial growth factor A	
NM_001295	CCR1	C-C motif chemokine receptor 1	Cytokine signaling
NM_000395	CSF2RB	colony stimulating factor 2 receptor	
NM_000584	CXCL8	C-X-C motif chemokine ligand 8	
NM_000601	HGF	hepatocyte growth factor	
NM_001547	IFIT2	interferon induced protein with	
NM_002188	IL13	interleukin 13	
NM_000575	IL1A	interleukin 1 alpha	
NM_021803	IL21	interleukin 21	
NM_000589	IL4	interleukin 4	
NM_000600	IL6	interleukin 6	
NM_000880	IL7	interleukin 7	
NM_003811	TNFSF9	tumor necrosis factor superfamily	
NM_005211	CSF1R	colony stimulating factor 1 receptor	
NM_000417	IL2RA	interleukin 2 receptor subunit alpha	
NM_003152	STAT5A	signal transducer and activator of	
NM_130441	CLEC4C	C-type lectin domain family 4 member	Dendridic cell
NM_017912	HERC6	HECT and RLD domain containing E3	
NM_002183	IL3RA	interleukin 3 receptor subunit alpha	
NM_000887	ITGAX	integrin subunit alpha X	
NM_003873	NRP1	neuropilin 1	
NM_003265	TLR3	toll like receptor 3	
NM_025224	ZBTB46	zinc finger and BTB domain containing	
NM_000591	CD14	CD14 molecule	Dendridic cell,macrophage
NM_021155	CD209	CD209 molecule	
NM_001142345	CMKLR1	chemerin chemokine-like receptor 1	
NM_052872	IL17F	interleukin 17F	
NM_016584	IL23A	interleukin 23 subunit alpha	
NM_002438	MRC1	mannose receptor C type 1	
NM_001242	CD27	CD27 molecule	Drug target
NM_001250	CD40	CD40 molecule	
NM_001252	CD70	CD70 molecule	
NM_005214	CTLA4	cytotoxic T-lymphocyte associated	
NM_002164	IDO1	indoleamine 23-dioxygenase 1	
NM_000572	IL10	interleukin 10	
NM_000882	IL12A	interleukin 12A	
NM_002187	IL12B	interleukin 12B	
NM_000586	IL2	interleukin 2	
NM_014218	KIR2DL1	killer cell immunoglobulin like receptor	
NM_002262	KLRD1	killer cell lectin like receptor D1	
NM_002286	LAG3	lymphocyte activating 3	
NM_021950	MS4A1	membrane spanning 4-domains A1	

NM_005018	PDCD1	programmed cell death 1	
NM_006928	PMEL	premelanosome protein	
NM_021181	SLAMF7	SLAM family member 7	
NM_139276	STAT3	signal transducer and activator of	
NM_017442	TLR9	toll like receptor 9	
NM_004195	TNFRSF18	tumor necrosis factor receptor	
NM_003327	TNFRSF4	tumor necrosis factor receptor	
NM_001561	TNFRSF9	tumor necrosis factor receptor	
NM_006399	BATF	basic leucine zipper ATF-like	Helper T cells
NM_001123396	CCR2	C-C motif chemokine receptor 2	
NM_000616	CD4	CD4 molecule	
NM_001002295	GATA3	GATA binding protein 3	
NM_002190	IL17A	interleukin 17A	
NM_005060	RORC	RAR related orphan receptor C	
NM_003151	STAT4	signal transducer and activator of	
NM_003153	STAT6	signal transducer and activator of	
NM_001025091	ABCF1	ATP binding cassette subfamily F	Housekeeping
NM_000402	G6PD	glucose-6-phosphate dehydrogenase	
NM_000181	GUSB	glucuronidase beta	
NM_000190	HMBS	hydroxymethylbilane synthase	
NM_170707	LMNA	lamin A C	
NM_002332	LRP1	LDL receptor related protein 1	
NM_000937	POLR2A	polymerase RNA II subunit A	
NM_004168	SDHA	succinate dehydrogenase complex	
NM_003194	TBP	TATA-box binding protein	
NM_001128148	TFRC	transferrin receptor	
NM_178014	TUBB	tubulin beta class I	
NM_021913	AXL	AXL receptor tyrosine kinase	Innate immune response
NM_015991	C1QA	complement component 1 q	
NM_007329	DMBT1	deleted in malignant brain tumors 1	
NM_022168	IFIH1	interferon induced with helicase C	
NM_000625	NOS2	nitric oxide synthase 2	
NM_016562	TLR7	toll like receptor 7	
NM_000491	C1QB	complement component 1 q	
NM_005564	LCN2	lipocalin 2	
NM_000239	LYZ	lysozyme	
NM_139012	MAPK14	mitogen-activated protein kinase 14	
NM_002415	MIF	macrophage migration inhibitory	
NM_014314	DDX58	DEXD H-box helicase 58	Interferon signaling
NM_005533	IFI35	interferon induced protein 35	
NM_006820	IFI44L	interferon induced protein 44 like	
NM_022873	IFI6	interferon alpha inducible protein 6	
NM_002460	IRF4	interferon regulatory factor 4	
NM_001178046	MX1	MX dynamin like GTPase 1	
NM_016817	OAS2	2-5-oligoadenylate synthetase 2	

NM_006187	OAS3	2-5-oligoadenylate synthetase 3	
NM_001081637	LILRB1	leukocyte immunoglobulin like	Leukocyte inhibition
NM_007161	LST1	leukocyte specific transcript 1	
NM_002209	ITGAL	integrin subunit alpha L	Leukocyte migration
NM_001145808	ITGAM	integrin subunit alpha M	
NM_000889	ITGB7	integrin subunit beta 7	
NM_000655	SELL	selectin L	
NM_001078	VCAM1	vascular cell adhesion molecule 1	
NM_002351	SH2D1A	SH2 domain containing 1A	Lymphocyte activation
NM_053282	SH2D1B	SH2 domain containing 1B	
NM_006060	IKZF1	IKAROS family zinc finger 1	Lymphocyte development
NM_016260	IKZF2	IKAROS family zinc finger 2	
NM_022465	IKZF4	IKAROS family zinc finger 4	
NM_002988	CCL18	C-C motif chemokine ligand 18	Lymphocyte infiltrate
NM_002982	CCL2	C-C motif chemokine ligand 2	
NM_002989	CCL21	C-C motif chemokine ligand 21	
NM_002983	CCL3	C-C motif chemokine ligand 3	
NM_002984	CCL4	C-C motif chemokine ligand 4	
NM_002985	CCL5	C-C motif chemokine ligand 5	
NM_001100168	CCR5	C-C motif chemokine receptor 5 gene	
NM_001767	CD2	CD2 molecule	
NM_001774	CD37	CD37 molecule	
NM_001803	CD52	CD52 molecule	
NM_001780	CD63	CD63 molecule	
NM_007074	CORO1A	coronin 1A	
NM_004079	CTSS	cathepsin S	
ENST00000399220	CX3CR1	C-X3-C motif chemokine receptor 1	
ENST00000435290	CX3CR1	C-X3-C motif chemokine receptor 1	
ENST00000541347	CX3CR1	C-X3-C motif chemokine receptor 1	
NM_001171174	CX3CR1	C-X3-C motif chemokine receptor 1	
NM_003467	CXCR4	C-X-C motif chemokine receptor 4	
NM_006564	CXCR6	C-X-C motif chemokine receptor 6	
NM_004106	FCER1G	Fc fragment of IgE receptor Ig	
NM_001465	FYB	FYN binding protein	
NM_006144	GZMA	granzyme A	
NM_004131	GZMB	granzyme B	
NM_033423	GZMH	granzyme H	
NM_002104	GZMK	granzyme K	
NM_005849	IGSF6	immunoglobulin superfamily member 6	
NM_001558	IL10RA	interleukin 10 receptor subunit alpha	
NM_000206	IL2RG	interleukin 2 receptor subunit gamma	
NM_000211	ITGB2	integrin subunit beta 2	
NM_001098526	JAML	junction adhesion molecule like	
NM_005561	LAMP1	lysosomal associated membrane	

NM_006762	LAPTM5	lysosomal protein transmembrane 5	
NM_001080978	LILRB2	leukocyte immunoglobulin like	
NM_002348	LY9	lymphocyte antigen 9	
NM_005601	NKG7	natural killer cell granule protein 7	
NM_001199797	PTPN7	protein tyrosine phosphatase non-	
NM_002838	PTPRC	protein tyrosine phosphatase receptor	
NM_015474	SAMHD1	SAM and HD domain containing	
NM_014450	SIT1	signaling threshold regulating	
NM_020125	SLAMF8	SLAM family member 8	
NM_002727	SRGN	serglycin	
NM_054114	TAGAP	T-cell activation RhoGTPase activating	
NM_001003806	TARP	TCR gamma alternate reading frame	
NM_138636	TLR8	toll like receptor 8	
NM_014350	TNFAIP8	TNF alpha induced protein 8	
NM_198125	TYROBP	TYRO protein tyrosine kinase binding	
NM_001623	AIF1	allograft inflammatory factor 1	
NM_001141	ALOX15B	arachidonate 15-lipoxygenase type B	
NM_004244	CD163	CD163 molecule	Macrophage
NM_001251	CD68	CD68 molecule	
NM_000569	FCGR3A	Fc fragment of IgG receptor IIIa	
NM_000045	ARG1	arginase 1	
NM_001772	CD33	CD33 molecule	
NM_001816	CEACAM8	carcinoembryonic antigen related cell	
NM_000250	MPO	myeloperoxidase	Myeloid marker
NM_002964	S100A8	S100 calcium binding protein A8	
NM_002965	S100A9	S100 calcium binding protein A9	
NM_002033	FUT4	fucosyltransferase 4	
NM_000717	CA4	carbonic anhydrase 4	
NM_032564	DGAT2	diacylglycerol O-acyltransferase 2	
NM_032045	KREMEN1	kringle containing transmembrane	Neutrophil
NM_052972	LRG1	leucine rich alpha-2-glycoprotein 1	
NM_002863	PYGL	phosphorylase glycogen liver	
NM_018644	B3GAT1	beta-13-glucuronyltransferase 1	
NM_000570	FCGR3B	Fc fragment of IgG receptor IIIb	
NM_006433	GNLY	granulysin	
NM_002258	KLRB1	killer cell lectin like receptor B1	
NM_016523	KLRF1	killer cell lectin like receptor F1	NK activation
NM_005810	KLRG1	killer cell lectin like receptor G1	
NM_007360	KLRK1	killer cell lectin like receptor K1	
NM_005041	PRF1	perforin 1	
ENST00000344867	KIR2DL2	killer cell immunoglobulin like receptor	
NM_015868	KIR2DL3	killer cell immunoglobulin like receptor	NK cell marker
NM_004829	NCR1	natural cytotoxicity triggering receptor	
NM_147130	NCR3	natural cytotoxicity triggering receptor	

NM_172387	NFATC1	nuclear factor of activated T-cells 1	PD-1 signaling
NM_001198	PRDM1	PR domain 1	
NM_002015	FOXO1	forkhead box O1	
NM_001530	HIF1A	hypoxia inducible factor 1 alpha	
NM_004958	MTOR	mechanistic target of rapamycin	
NM_006218	PIK3CA	phosphatidylinositol-45-bisphosphate	
NM_005026	PIK3CD	phosphatidylinositol-45-bisphosphate	
NM_000314	PTEN	phosphatase and tensin homolog	
NM_002834	PTPN11	protein tyrosine phosphatase non-	
NM_004336	BUB1	BUB1 mitotic checkpoint serine	Proliferation
NM_004701	CCNB2	cyclin B2	
NM_001786	CDK1	cyclin-dependent kinase 1	
NM_005192	CDKN3	cyclin-dependent kinase inhibitor 3	
NM_021953	FOXM1	forkhead box M1	
NM_014736	KIAA0101	KIAA0101	
NM_002358	MAD2L1	MAD2 mitotic arrest deficient-like 1	
NM_014791	MELK	maternal embryonic leucine zipper	
NM_002417	MKI67	marker of proliferation Ki-67	
NM_001067	TOP2A	topoisomerase DNA II alpha	
NM_000399	EGR2	early growth response 2	T cell differentiation
NM_001110533	LEXM	lymphocyte expansion molecule	
NM_170662	CBLB	Cbl proto-oncogene B	T cell receptor signaling
NM_000074	CD40LG	CD40 ligand	
NM_021268	IFNA17	interferon alpha 17	
NM_020529	NFKBIA	NFKB inhibitor alpha	
NM_080548	PTPN6	protein tyrosine phosphatase non-	
NM_001079	ZAP70	zeta chain of T cell receptor associated	
NM_005755	EBI3	Epstein-Barr virus induced 3	T cell regulation
NM_014009	FOXP3	forkhead box P3	
NM_002166	ID2	inhibitor of DNA binding 2 HLH protein	
NM_002167	ID3	inhibitor of DNA binding 3 HLH protein	
NM_000585	IL15	interleukin 15	
NM_001562	IL18	interleukin 18	
NM_020525	IL22	interleukin 22	
NM_002355	M6PR	mannose-6-phosphate receptor cation	
NM_016270	KLF2	Kruppel like factor 2	
NM_001838	CCR7	C-C motif chemokine receptor 7	
NM_198053	CD247	CD247 molecule	
NM_000732	CD3D	CD3d molecule	
NM_000733	CD3E	CD3e molecule	
NM_000073	CD3G	CD3g molecule	
NM_006725	CD6	CD6 molecule	
NM_171827	CD8A	CD8a molecule	

NM_172213	CD8B	CD8b molecule	TCR coexpression
NM_019604	CRTAM	cytotoxic and regulatory T-cell	
NM_001098200	GPR18	G protein-coupled receptor 18	
NM_004810	GRAP2	GRB2-related adaptor protein 2	
NM_012481	IKZF3	IKAROS family zinc finger 3	
NM_000878	IL2RB	interleukin 2 receptor subunit beta	
NM_002185	IL7R	interleukin 7 receptor	
NM_005546	ITK	IL2 inducible T-cell kinase	
NM_014398	LAMP3	lysosomal associated membrane	
NM_001042771	LCK	LCK proto-oncogene Src family tyrosine	
ENST00000326294	PTPRCAP	protein tyrosine phosphatase receptor	
NM_173799	TIGIT	T-cell immunoreceptor with Ig and ITIM	
NM_182482	BAGE	B melanoma antigen	Tumor antigen
NM_001327	CTAG1B	cancer testis antigen 1B	
ENST00000369585	CTAG2	cancer testis antigen 2	
NM_001040663	GAGE1,GAGE12	G antigen 1	
NM_001098413	GAGE10	G antigen 10	
NM_001098406	GAGE12J	G antigen 12J	
NM_001098412	GAGE13	G antigen 13	
NM_001472	GAGE2C,GAGE2	G antigen 2C	
NM_004988	MAGEA1	MAGE family member A1	
NM_021048	MAGEA10	MAGE family member A10	
NM_005367	MAGEA12	MAGE family member A12	
NM_005362	MAGEA3	MAGE family member A3	
NM_001011548	MAGEA4	MAGE family member A4	
NM_016249	MAGEC2	MAGE family member C2	
NM_005511	MLANA	melan-A	
NM_003147	SSX2	SSX family member 2	
NM_001097594	XAGE1B	X antigen family member 1B	
NM_001014431	AKT1	AKT serine threonine kinase 1	Tumor marker
NM_007300	BRCA1	BRCA1 DNA repair associated	
NM_000059	BRCA2	BRCA2 DNA repair associated	
NM_000077	CDKN2A	cyclin-dependent kinase inhibitor 2A	
NM_005227	EFNA4	ephrin A4	
NM_005228	EGFR	epidermal growth factor receptor	
NM_004430	EGR3	early growth response 3	
NM_005544	IRS1	insulin receptor substrate 1	
NM_000424	KRT5	keratin 5	
NM_005556	KRT7	keratin 7	
NM_002745	MAPK1	mitogen-activated protein kinase 1	
NM_004530	MMP2	matrix metallopeptidase 2	
NM_004994	MMP9	matrix metallopeptidase 9	
NM_002467	MYC	v-myc avian myelocytomatosis viral	
NM_000435	NOTCH3	notch 3	
NM_002632	PGF	placental growth factor	
NM_000963	PTGS2	prostaglandin-endoperoxide synthase	
NM_002821	PTK7	protein tyrosine kinase 7 inactive	

NM_000321	RB1	RB transcriptional corepressor 1	
NM_001010	RPS6	ribosomal protein S6	
NM_003202	TCF7	transcription factor 7 T-cell specific	
NM_003722	TP63	tumor protein p63	
NM_012101	TRIM29	tripartite motif containing 29	
NM_005985	SNAI1	snail family transcriptional repressor 1	
NM_003068	SNAI2	snail family transcriptional repressor 2	
NM_000474	TWIST1	twist family bHLH transcription factor 1	
NM_001174093	ZEB1	zinc finger E-box binding homeobox 1	
NM_004335	BST2	bone marrow stromal cell antigen 2	Type I interferon signaling
NM_005532	IFI27	interferon alpha inducible protein 27	
NM_001548	IFIT1	interferon induced protein with	
NM_001031683	IFIT3	interferon induced protein with	
NM_003641	IFITM1	interferon induced transmembrane	
NM_006435	IFITM2	interferon induced transmembrane	
NM_005101	ISG15	ISG15 ubiquitin-like modifier	
NM_002201	ISG20	interferon stimulated exonuclease	
NM_001706	BCL6	B-cell CLL lymphoma 6	Type II interferon signaling
NM_000246	CIITA	class II major histocompatibility	
NM_002996	CX3CL1	C-X3-C motif chemokine ligand 1	
NM_001565	CXCL10	C-X-C motif chemokine ligand 10	
NM_005409	CXCL11	C-X-C motif chemokine ligand 11	
NM_006419	CXCL13	C-X-C motif chemokine ligand 13	
NM_002416	CXCL9	C-X-C motif chemokine ligand 9	
NM_001716	CXCR5	C-X-C motif chemokine receptor 5	
NM_000397	CYBB	cytochrome b-245 beta chain	
NM_001135651	EIF2AK2	eukaryotic translation initiation factor	
NM_000639	FASLG	Fas ligand	
NM_002053	GBP1	guanylate binding protein 1	
NM_000201	ICAM1	intercellular adhesion molecule 1	
NM_002176	IFNB1	interferon beta 1	
NM_000619	IFNG	interferon gamma	
NM_000576	IL1B	interleukin 1 beta	
NM_002198	IRF1	interferon regulatory factor 1	
NM_006084	IRF9	interferon regulatory factor 9	
NM_016816	OAS1	2-5-oligoadenylate synthetase 1	
NM_002800	PSMB9	proteasome subunit beta 9	
NM_007315	STAT1	signal transducer and activator of	
NM_000593	TAP1	transporter 1 ATP-binding cassette sub-	
NM_013351	TBX21	T-box 21	
